# The Role of Dihydroorotate Dehydrogenase in Apoptosis Induction in Response to Inhibition of the Mitochondrial Respiratory Chain Complex III

**Published:** 2014

**Authors:** A. A. Khutornenko, A. A. Dalina, B. V. Chernyak, P. M. Chumakov, A. G. Evstafieva

**Affiliations:** Belozersky Institute of Physico-Chemical Biology, Lomonosov Moscow State University, Leninskie Gory, 1, Bld. 40, 119991, Moscow, Russia; Department of Bioengineering and Bioinformatics, Lomonosov Moscow State University, Leninskie Gory, 1, Bld. 73, 119991, Moscow, Russia; Engelhardt Institute of Molecular Biology, Russian Academy of Sciences, Vavilova Str., 32, 119991, Moscow, Russia.

**Keywords:** apoptosis, p53 tumor suppressor, mitochondrial respiratory chain, dihydroorotate dehydrogenase, de novo pyrimidine biosynthesis

## Abstract

A mechanism for the induction of programmed cell death (apoptosis) upon dysfunction of the mitochondrial respiratory chain has been studied. Previously, we had found that inhibition of mitochondrial cytochrome* bc*1, a component of the electron transport chain complex III, leads to activation of tumor suppressor p53, followed by apoptosis induction. The mitochondrial respiratory chain is coupled to the *de novo *pyrimidine biosynthesis pathway via the mitochondrial enzyme dihydroorotate dehydrogenase (DHODH). The p53 activation induced in response to the inhibition of the electron transport chain complex III has been shown to be triggered by the impairment of the *de novo *pyrimidine biosynthesis due to the suppression of DHODH. However, it remained unclear whether the suppression of the DHODH function is the main cause of the observed apoptotic cell death. Here, we show that apoptosis in human colon carcinoma cells induced by the mitochondrial respiratory chain complex III inhibition can be prevented by supplementation with uridine or orotate (products of the reaction catalyzed by DHODH) rather than with dihydroorotate (a DHODH substrate). We conclude that apoptosis is induced in response to the impairment of the *de novo *pyrimidine biosynthesis caused by the inhibition of DHODH. The conclusion is supported by the experiment showing that downregulation of DHODH by RNA interference leads to accumulation of the p53 tumor suppressor and to apoptotic cell death.

## INTRODUCTION

Mitochondria play a central role in homeostasis in eukaryotic cells. They both supply the cell with energy by means of oxidative phosphorylation and act as important mediators of programmed cell death, as well as of the intracellular signaling cascades mediated by calcium ions and reactive oxygen species [15]. The mitochondrial respiratory chain (MRC ) consists of multicomponent I-IV protein complexes integrated into the inner mitochondrial membrane, which catalyze electron transfer from NADH to molecular oxygen. This leads to the generation of the electrochemical proton gradient through the inner mitochondrial membrane, which is the driving force behind ATP synthesis by means of ATP synthase (complex V).

Many human diseases are associated with mitochondrial dysfunctions; moreover, the so-called “mitochondrial diseases” are usually caused by respiratory chain defects in these organelles [2]. Mitochondrial dysfunctions are involved in the aging process [3]. With age, the number of mutations in mammalian mitochondrial DNA increases and respiratory chain dysfunction is observed. Cells with defects in the MRC are prone to apoptosis, and the increased cell loss is an important consequence of mitochondrial dysfunctions. In this paper we address the mechanism of apoptotic program activation upon MRC dysfunction.

The tumor suppressor p53 is a key regulatory protein that in many cases determines cell behavior in different types of stress: whether cell-cycle arrest occurs, accompanied by damage repair, or mechanisms of programmed cell death are activated, which are aimed at deleting cells with unrepairable damage [4]. Previously, we had found that the inhibition of the MRC complex III leads to an increase in the level of p53 and its activity, as well as to the activation of programmed death of human cancer cells [5]. The p53 activation appeared to be caused not by the inhibition of the MRC itself, but by the dysfunction of complex III (complex of cytochrome* bc*1) that transfers electrons from reduced ubiquinone (ubiquinol) to cytochrome *c*. One of the most important metabolic pathways in the cell, the *de novo *pyrimidine biosynthesis is coupled with the MRC [6]. The only mitochondrial enzyme of this pathway is dihydroorotate dehydrogenase (DHODH), which oxidizes dihydroorotate to orotate and uses ubiquinone as an electron acceptor [6]. The dysfunction of MRC complex III results in the transition of ubiquinone to the reduced state, which in turn may inhibit the DHODH function and lead to impairment of pyrimidine biosynthesis. Indeed, we demonstrated that an increase in the level and activity of p53 upon inhibition of the MRC complex III is due to the impairment of the DHODH function and *de novo *pyrimidine biosynthesis [5]. However, it remained unclear whether the suppression of the DHODH function is the main reason behind the activation of the cell apoptotic program upon inhibition of MRC complex III.

In this paper, we have demonstrated that impairment of the DHODH function and, as a consequence, of *de novo *pyrimidine biosynthesis induces apoptosis in human colon cancer cells upon inhibition of MRC complex III.

## EXPERIMENTAL

**Conditions for cell culturing and treatment**

RKO and HCT 116 human colon cancer cells were grown on a DMEM medium supplemented with 10% fetal bovine serum (HyClone) at 37 0C and 5% CO_2_ to 50–70% confluency. Then, the cells were incubated for 12 h to determine the p53 level and for 20–26 h to analyze apoptosis in the presence of 200 nM myxothiazol (Sigma-Aldrich Inc.). In some experiments, the medium was supplemented with uridine to a final concentration of 50 μg/ml; orotate or dihydroorotate (Sigma-Aldrich Inc.) to a final concentration of 1 mM.

**Evaluation of apoptosis by flow cytometry**

The cells were detached from the scaffold by tryptic cleavage, washed with phosphate-buffered saline (PBS, 0.14 M NaCl; 2.7 mM KCl; 10 mM Na_2_HPO_4_; 1.8 mM KH_2_PO_4_, pH 7.3), and suspended in 100 μl of a Annexin buffer (10 mM HEPES; 140 mM NaCl; 2.5 mM CaCl_2_, pH 7.4). Then the cells were supplemented with 7.5 μl of Annexin V conjugated to FITC (Invitrogen) and with propidium iodide (Clontech) to a final concentration of 100 μg/ml and incubated in the dark for 15 min. Thereafter, another 500 μl of the Annexin buffer was added; the cell suspension was filtered through a 30 μm filter and analyzed on a Partec PASIII flow cytometer.

**Immunoblotting**

The cells were lysed in a RLB buffer (Promega Inc.). Equal amounts of protein extracts (50–100 μg) were fractionated by electrophoresis in 12% SDS-PAGE; electrotransfer of the proteins onto a nitrocellulose membrane and treatment of the membrane were performed as previously described [7]. The membrane was incubated with mouse monoclonal antibodies to DHODH (ab54621, Abcam), to p53 (DO-1), or to actin (G-2) (Santa Cruz Biotechnology Inc.) diluted at a ratio of 1 : 500 with a TBST buffer (20 mM Tris-HCl, pH 7.5; 140 mM NaCl; 0.05% Tween-20) for 2 h. To control loading, the membranes were incubated with actin antibodies. Detection was performed using secondary sheep anti-mouse antibodies conjugated to horseradish peroxidase (GE Healthcare) and enhanced chemiluminescence according to the standard technique (Western Lightning Chemiluminescence Reagent, Perkin Elmer Life Sciences).

**Preparation of cell lines with a reduced DHODH level**

Lentiviral vectors based on the pLKO.1-puro plasmid (Sigma-Aldrich Inc.) contained the genes of short hairpin RN As to DHODH with the following sequences: si21 – CC GGTCC GGGATTT ATC AACTC AAACTC - GAGTTT GA GTT GATAAATCCC GGATTTTT , si32 – CC GGCGGACTTT ATAAGATGGGCTTCTC GA GAAGCCC ATCTT ATAAAGTCC G TTTTT .

For each lentiviral vector, pLKO-si21 and pLKOsi32, viral stocks were obtained. For this purpose, HEK293T human embryonic kidney cells on 10-cm Petri dishes were transfected with the corresponding lentiviral vector and a set of packaging plasmids [8] using LipofectAMIN 2000 (Invitrogen) according to the manufacturer’s procedure. A mixture of four plasmids was used: the 3 μg lentiviral vector, 12 μg plasmid pRev2 expressing the protein Rev, 6 μg plasmid pGag1 expressing the proteins Pol and Gag, and 3 μg plasmid pVSV-G expressing glycoprotein G of the vesicular stomatitis virus (a total of 24 μg DNA). The plasmid mixture, diluted with the DMEM medium, was mixed with the diluted LipofectAMIN 2000 (60 μL), stirred vigorously, incubated for 20 min at room temperature, and pipetted into a plate with the cells. On the next day, the medium was replaced with 10 ml of DMEM containing 2% fetal bovine serum.

The secreted viral particles were harvested 2 days after transfection: 10 ml of the medium from the transfected cells was filtered through a low protein binding filter (Durapore membrane, Millex-HV, Millipore) with 0.45 μm pores; 1 ml aliquots were stored at –70 °C.

RKO cells were infected with viral particles carrying two different variants of the gene of short hairpin RN A to DHODH (si32 and si21), as well as with control viruses containing no short hairpin RN A (pLS-Lpw).

For this purpose, cells grown on 35-mm plates were supplemented with 1 ml of viral particles diluted in 1 ml of a fresh medium and 5–8 μg of polybrene (Hexadimethrine bromide, Sigma-Aldrich Inc.). The cells were grown in the presence of uridine (50 μg/ml). Three days later, puromycin was added (1 μg/ml) and the selection was conducted for 3 more days. The cells were lysed; the DHODH level was determined using immunoblotting.

## RESULTS

**The role of the impairment of pyrimidine biosynthesis in apoptosis induction upon the inhibition of the mitochondrial respiratory chain complex III**

**Fig. 1:**
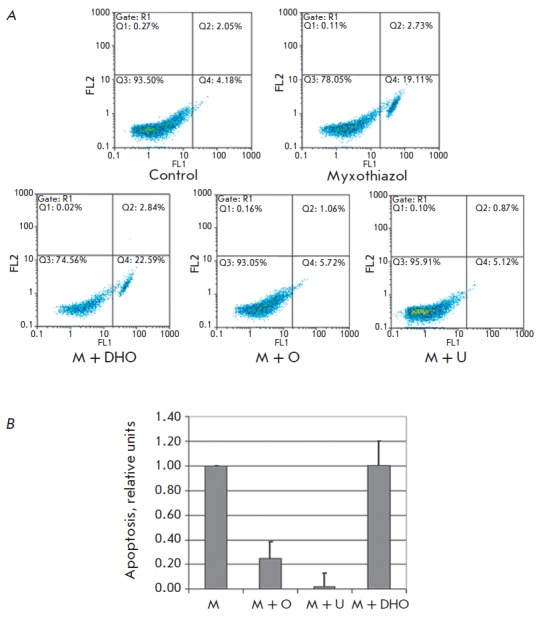
Uridine and orotate, but not dihydroorotate, protect RKO cells against myxothiazol-induced apoptosis. The apoptosis level of RKO cells stained with FITC-Annexin V and PrI was determined by flow cytometry. *A *– a representative 2D diagram of cell distribution over the fluorescence intensity in FL2 (PrI) and FL1 (FITC-Annexin V) channels. Cells were analyzed 26 h after treatment with 200 nM myxothiazol (Myxothiazol, M) only or along with 1 mM dihydroorotate (M + DHO), 1 mM orotate (M + O), and 1 mM uridine (M + U). The control is untreated cells. *B – *Statistical analysis of the results. Percentage of apoptotic (AnnexinV-positive, PrI-negative) cells in each sample after subtraction of control values was normalized to the percentage of cells in which apoptosis was induced by myxothiazol without additives. The diagram shows the mean values of the relative apoptosis level and SDs of three independent experiments

We have shown that the action of the MRC complex III inhibitors leads to growth arrest in a number of cell lines of epithelial tumors and to their massive death. A cytometric analysis of RKO human colon cancer cells treated with myxothiazol, an inhibitor of MRC complex III, and stained with FITC -Annexin V and propidium iodide (PrI) revealed a significant population of apoptotic cells (*Fig. 1*). In general, three cell populations were observed: normal cells (Annexin V-negative, PrI-negative), apoptotic cells (Annexin V-positive, PrInegative, approximately 20% of all cells), and a third small population of dead cells (Annexin V-positive, PrI-positive, whose fraction was approximately 3%). The size of the third population was bigger when cells were collected not only from the scaffold, but from the medium as well (data not shown), and these cells were considered as necrotic or late-apoptotic.

Since the mitochondrial respiratory chain is functionally coupled with the *de novo *pyrimidine biosynthesis pathway via the dihydroorotate dehydrogenase inserted in the mitochondrial membrane [6], we decided to test how replenishment of the pyrimidine pool affects myxothiazol-induced apoptosis. For this purpose, a cytometric analysis was performed after treatment of RKO cells with myxothiazol in the presence of uridine. Uridine, a precursor of both uridylic and cytidylic nucleotides, appeared to almost completely prevent the accumulation of apoptotic Annexin Vpositive, PrI-negative cells caused by treatment with myxothiazol (*Fig. 1*). This indicates that the reason for apoptosis induction is impairment of the *de novo* pyrimidine biosynthesis, presumably due to DHODH inhibition.

To directly assess the role of DHODH, RKO cells were treated with myxothiazol in the presence of a substrate or a product of the DHODH-catalyzed reaction; the apoptosis level was analyzed by flow cytometry. Dihydroorotate (a DHODH substrate) had no effect on myxothiazol-induced apoptosis (*Fig. 1*), but orotate (a product of the DHODH-catalyzed reaction) substantially prevented it (the number of apoptotic cells was 4 times lower than upon apoptosis induction by myxothiazol (*Fig. 1*).

Similar results were obtained for the other human colon cancer cell line, HCT 116 (not shown).

The obtained data suggest that apoptosis induction upon inhibition of MRC complex III is, to a great extent, due to the DHODH inhibition and impairment of the *de novo *pyrimidine biosynthesis. For more confidence in this molecular mechanism, it was decided to conduct the reverse experiment and to check whether dysfunction of DHODH causes apoptotic cell death similarly to the inhibition of MRC complex III.

**The effect of dihydroorotate dehydrogenase knockdown on tumor suppressor p53 and programmed cell death**

**Fig. 2:**
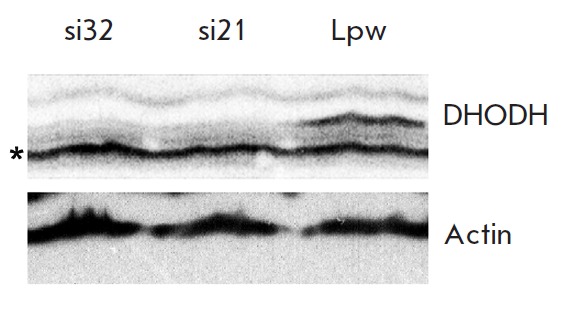
Efficiency of DHODH-specific RNA interference. Western blot analysis of DHODH levels in the lysates of RKO cells infected with pLKO-si21 (si21), or with pLKOsi32 (si32), or with the empty vector pLS-Lpw (Lpw). The upper panel shows the reaction with DHODH antibodies, the lower panel shows the reaction with β-actin antibodies used as a loading control. The asterisk (*) denotes a nonspecific band, which can also serve as a sample loading control

Does dysfunction of DHODH really cause apoptotic cell death similarly to the inhibition of the MRC complex III? To find out, it was decided to prepare a RKO cell line with DHODH expression suppressed by RN A interference. The lentiviral system was used for effective delivery of a cassette expressing short interfering RN As. RKO cells were infected with the lentiviral particles carrying two different variants of the gene of short hairpin RN A to DHODH (si32 and si21) and also with the control viruses, which did not contain these genes (pLS-Lpw) and were grown in the presence of uridine. The cells with expression cassettes integrated into the chromosome were selected using puromycin and lysed; the DHODH level was determined using immunoblotting (*Fig. 2*).

Thus, the DHODH level in cells expressing two different short hairpin RN As to DHODH was found to be significantly lower than in cells infected with viral particles on the basis of the “empty” vector (*Fig. 2*).

**Fig. 3:**
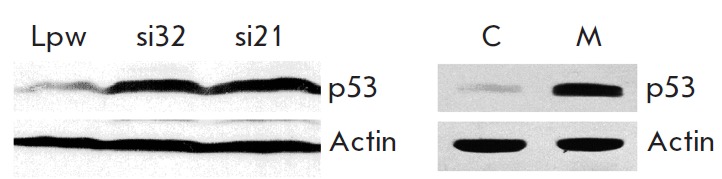
DHODH interference in RKO cells results in p53 induction similarly to the effect of myxothiazol, the inhibitor of the MRC complex III. Western blot analysis of p53 levels in RKO cells infected with pLKO-si21 (si21), or with pLKO-si32 (si32), or with the empty vector pLS-Lpw (Lpw). Cells were cultured in the absence of uridine for 24 h. For comparison, the right panel shows the Western blot analysis of p53 levels in RKO cells untreated (C) or treated with 200 nM myxothiazol (M) for 12 h. Upper panel –with p53 antibodies; lower panel – with β-actin antibodies

Previously, we had shown that the inhibition of MRC complex III leads to activation of the tumor suppressor p53 due to the dysfunction of DHODH [5]. To determine whether DHODH knockdown causes accumulation of p53, immunoblotting was used to compare the p53 level in the control cells and the cells with RN A interference specific to DHODH, cultured in the absence of an external uridine source. The p53 level in the cells with DHODH knockdown appeared to increase in the same way as the inhibition of the MRC complex III (*Fig. 3*).

**Fig. 4:**
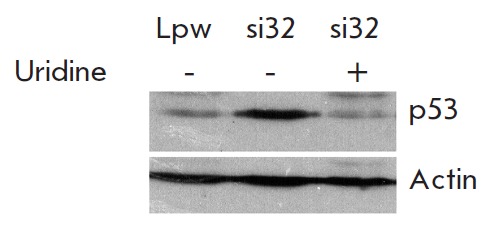
Uridine prevents p53 induction in cells with DHODH knockdown. Western blot analysis of p53 levels in RKO cells infected with pLKO-si32 (si32) or with the empty vector pLS-Lpw (Lpw). Cells were cultured in the absence (–) or in the presence (+) of uridine for 24 h. Upper panel – with p53 antibodies; lower panel – with β-actin antibodies

Uridine prevented the accumulation of p53 in cells with RN A interference specific to DHODH (*Fig. 4*). Consequently, impairment of the *de novo *pyrimidine biosynthesis can be considered as the most likely cause of the increased p53 level in these cells.

**Fig. 5:**
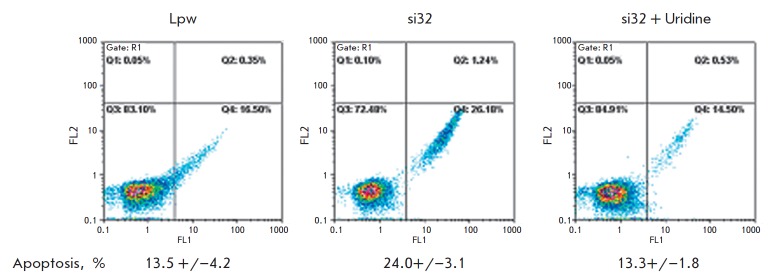
Uridine protects RCO cells with DHODH knockdown against apoptosis. The apoptosis level of RKO cells, either control (Lpw) or with DHODH-specific RNA interference, cultured in the absence of an external uridine source (si32) or in the presence of uridine (si32 + uridine), was measured by flow cytometry. Cells were stained with FITC-Annexin V and propidium iodide (PrI). Results are presented as a 2D diagram of cell distribution over the fluorescence intensity in the FL2 (PrI) and FL1 (FITC-Annexin V) channels. The bottom panel shows the percentage of apoptotic (AnnexinV-positive, PrI-negative) cells (the mean value +/– SD of three independent experiments)

Further, a cytometric analysis was performed for FITC -Annexin V and propidium iodide-stained cells with RN A interference specific to DHODH, which were cultured in the absence of an external uridine source. It turned out that an increase in the fraction of apoptotic Annexin V-positive, PrI-negative cells is the functional consequence of the suppression of DHODH expression and stabilization of p53 (*Fig. 5*). Adding uridine to the growth medium reduced the percentage of apoptotic cells to the reference level, which proves the specificity of the observed effect.

Thus, it was demonstrated by suppressing DHODH expression using the RN A interference method that both the dysfunction of DHODH and the inhibition of MRC complex III lead to elevation of the intracellular level of tumor suppressor p53 and to an increase in the level of programmed cell death (apoptosis). These results support our model, according to which apoptosis induction upon inhibition of the MRC complex III, as well as activation and stabilization of p53, occurs due to DHODH inhibition and impairment of *de novo *pyrimidine biosynthesis.

## DISCUSSION

Mitochondria are the “power stations” of the cell and, simultaneously, mediators of a number of regulatory pathways, including apoptosis induction [5]. Previously, we had demonstrated that the inhibition of the MRC complex III leads to the activation of tumor suppressor p53 and to the triggering of the cell death program [5]. The activation of p53 turned out to be caused not by the electron transport chain inhibition itself, but by the dysfunction of the cytochrome *bc*1 complex. It was demonstrated that this occurs due to the inhibition of dihydroorotate dehydrogenase, the only mitochondrial enzyme of the *de novo *pyrimidine biosynthesis pathway. However, it remained unknown whether the inhibition of DHODH was the exclusive cause behind the triggering of programmed cell death upon inhibition of the MRC complex III.

DHODH is a flavoprotein inserted in the inner mitochondrial membrane. DHODH oxidizes dihydroorotate to orotate and uses ubiquinone as an electron acceptor [6]. This paper demonstrates that apoptotic cell death induced by myxothiazol (an inhibitor of the MRC complex III) is fully prevented by uridine (a precursor of uridylic and cytidylic nucleotide biosynthesis) and to a large extent by orotat (a product of the reaction catalyzed by dihydroorotate dehydrogenase). Meanwhile, dihydroorotate, a DHODH substrate, does not possess this property. These data suggest that apoptotic cell death upon inhibition of the MRC complex III is in reality caused by the inhibition of DHODH, the mitochondrial enzyme of the *de novo *pyrimidine biosynthesis pathway. This conclusion is confirmed by the results of experiments on the suppression of DHODH expression using RN A interference. DHODH knockdown turned out to result in the accumulation of tumor suppressor p53 and acceleration of apoptosis.

**Fig. 6:**
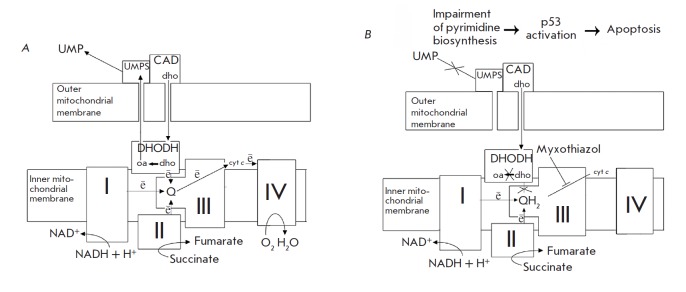
A model explaining the proposed mechanism of induction of p53-dependent apoptosis in response to MRC complex III inhibition. I, II, III, IV – the MRC complexes; Q – ubiquinone; QH2 – ubiquinol; cyt *c *– cytochrome *c*; DHODH – dihydroorotate dehydrogenase; dho – dihydroorotate; oa – orotate; UMPS – uridine monophosphate synthase, UMP – uridine monophosphate, CAD – multifunction enzyme that initiates the *de novo *pyrimidine biosynthesis, OMM and IMM – outer and inner mitochondrial membranes: electrons are shown as *e. A *–untreated cells, *B *– after treatment with myxothiazol. Explanations are provided in the text

*Figure 6 *provides a tentative diagram of the events resulting in apoptosis upon inhibition of the MRC complex III. Under normal conditions, ubiquinone accepts electrons from complex I, complex II, and dihydroorotate dehydrogenase. At that, ubiquinone is reduced to ubiquinol, which then donates electrons to cytochrome* c *through complex III (*Fig. 6A*). Myxothiazol-induced inhibition of MRC complex III blocks ubiquinol oxidation; ubiquinone passes to the completely reduced state and loses its ability to accept electrons during dihydroorotate oxidation. This leads to dysfunction of DHODH and, as a consequence, to impairment of *de novo *pyrimidine biosynthesis, stabilization and activation of tumor suppressor p53, and induction of programmed cell death (*Fig. 6B*). The significance of ubiquinone regeneration within the respiratory chain for* de novo *pyrimidine biosynthesis is confirmed by the fact that, as recently established, the malaria parasite* Plasmodium falciparum *apparently maintains the active mitochondrial electron transport chain exclusively for this purpose [9].

The results are in good agreement with data indicating that the DHODH inhibitor leflunomide/teriflunomide induces apoptosis in a number of human cancer cell lines [10-12]. However, according to [12], transformed keratinocytes with the mutant p53 gene, which lack transcriptionally active p53, are more sensitive to apoptosis induced by teriflunomide than normal keratinocytes with wild-type p53. In normal human epidermal keratinocytes (NHEK) a long-term exposure to teriflunomide was shown to induce cell cycle arrest at Go/G1 due to an induction of the p53 regulated gene CDKN1A encoding the cyclin-dependent kinase inhibitor p21. The response apparently reflects a cytoprotective role for p53 against teriflunomide-induced apoptosis [12]. Treatment of human fibroblasts with PALA, another inhibitor of pyrimidine biosynthesis (N-phosphonacetyl- L-aspartate, transcarbamylase inhibitor), led to reversible cell cycle arrest, survival of cells expressing transcriptionally active p53, and apoptotic cell death in the absence of p53 [13-15]. It is assumed that under conditions of suppressed pyrimidine biosynthesis, the cytoprotective properties of p53 (promoting the survival of normal cells with wild-type p53 and death of cancer cells with inactivated p53) may be used for anti-tumor therapy employing the proper inhibitors [12].

In contrast to the published data [12-15], the present work demonstrates that the suppression of DHODH activity and impairment of *de novo *pyrimidine biosynthesis lead to apoptosis induction in human colon cancer cells expressing transcriptionally active p53. Moreover, we had previously shown that HCT 116 p53^-/-^ cells (cells lacking p53) demonstrate significant suppression of apoptosis compared to wild-type HCT 116 cells [5]. Hence, in the studied tumor cells, p53 does not perform the cytoprotective function, but instead it promotes apoptosis induction upon impairment of *de novo *pyrimidine biosynthesis. The discrepancy between our results and the results of [12-15] may be due to tissue-specific variations and requires further study.

Our findings, as one of the consequences, suggest a possible application of inhibitors of pyrimidine biosynthesis in malignant human colon tumors expressing wild-type p53.

## CONCLUSIONS

The mechanism of programmed cell death activation upon dysfunction of the mitochondrial respiratory chain has been investigated. It has been demonstrated that dysfunction of the mitochondrial enzyme dihydroorotate dehydrogenase, leading to blockage of the* de novo *pyrimidine biosynthesis pathway, activation of tumor suppressor p53 and, as a result, induction of p53-dependent apoptosis is the reason behind apoptosis induction in human colon cancer cells upon inhibition of the mitochondrial respiratory chain complex III.

The results disagree with the previously published data, according to which tumor suppressor p53 in human keratinocytes and fibroblasts plays the cytoprotective role and protects cells against apoptosis induced by inhibitors of pyrimidine biosynthesis. We have demonstrated that suppression of DHODH activity and impairment of *de novo *pyrimidine biosynthesis induce apoptosis in human colon cancer cells expressing transcriptionally active p53. In the studied cell lines, p53, in contrast, promoted apoptosis induction upon impairment of *de novo *pyrimidine biosynthesis. The discrepancy between our results and previously published results may be due to tissue-specific variations and requires further study. The findings suggest a possible application of inhibitors of pyrimidine biosynthesis in human colon tumors expressing wild-type p53.

## Abbreviations

MRC: mitochondrial respiratory chain
DHODH: dihydroorotate dehydrogenase
shRNA: short hairpin RNA
SDS: sodium dodecyl sulfate
PAAG: polyacrylamide gel
PrI: propidium iodide
FITC: fluorescein isothiocyanate
